# Supplementary figures and images for: A network-based approach to identifying correlations between phylogeny, morphological traits and occurrence of fish species in US river basins
Source: PLoS One. 2023 Jun 23;18(6):e0287482. doi: 10.1371/journal.pone.0287482 (PMC10289417; doi:10.1371/journal.pone.0287482)

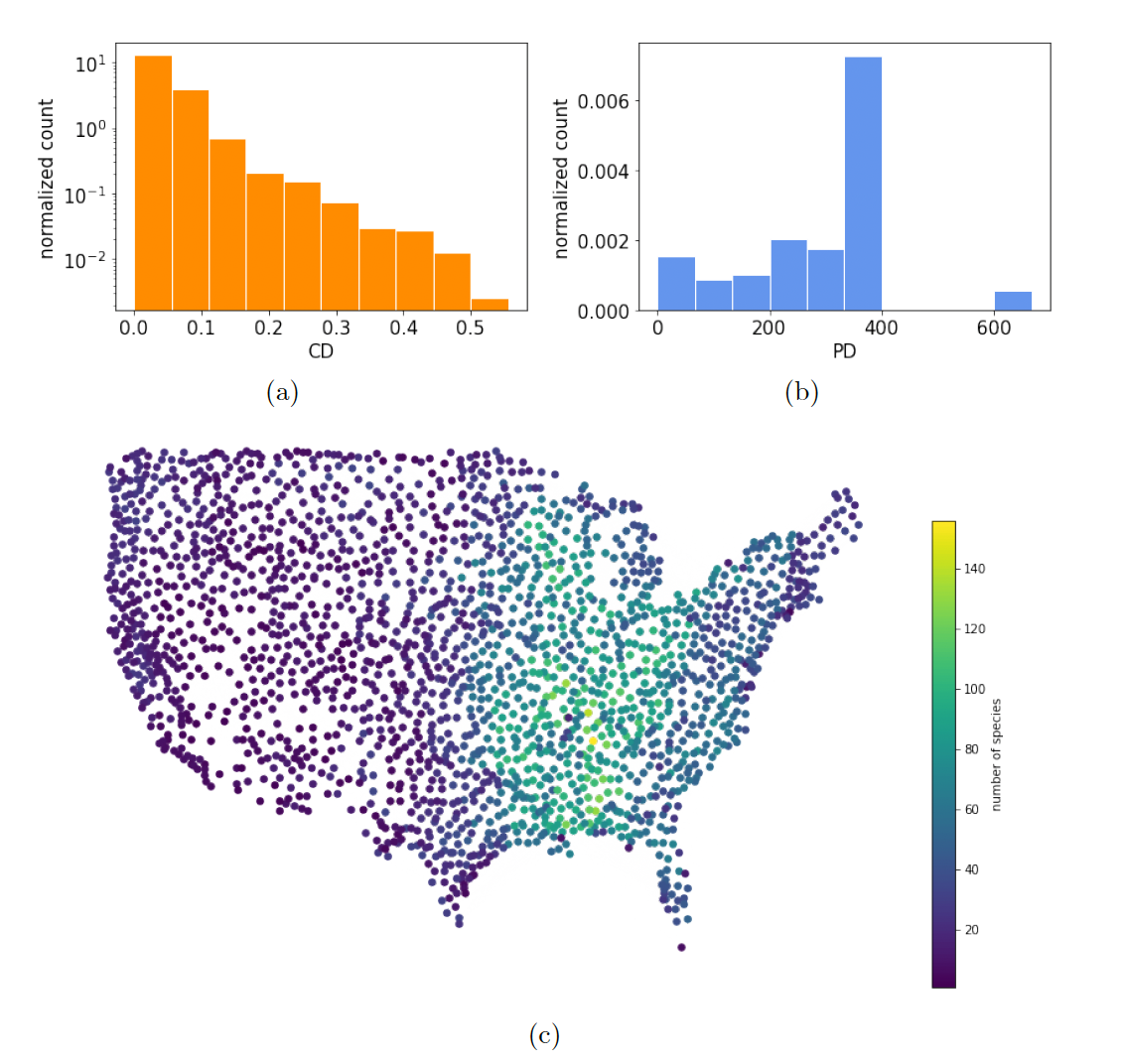

Supplement: S1 Fig — (a-b) Distribution of Phylogenetic distances and Cosine distances between fish species (c) Map of number of species in each basin: The dots indicate the centroids of HUC8 regions (obtained from USGS) and colours the number species present in the region. The range of species numbers is shown in the color map along side. (TIF) [file pone.0287482.s001.tif]

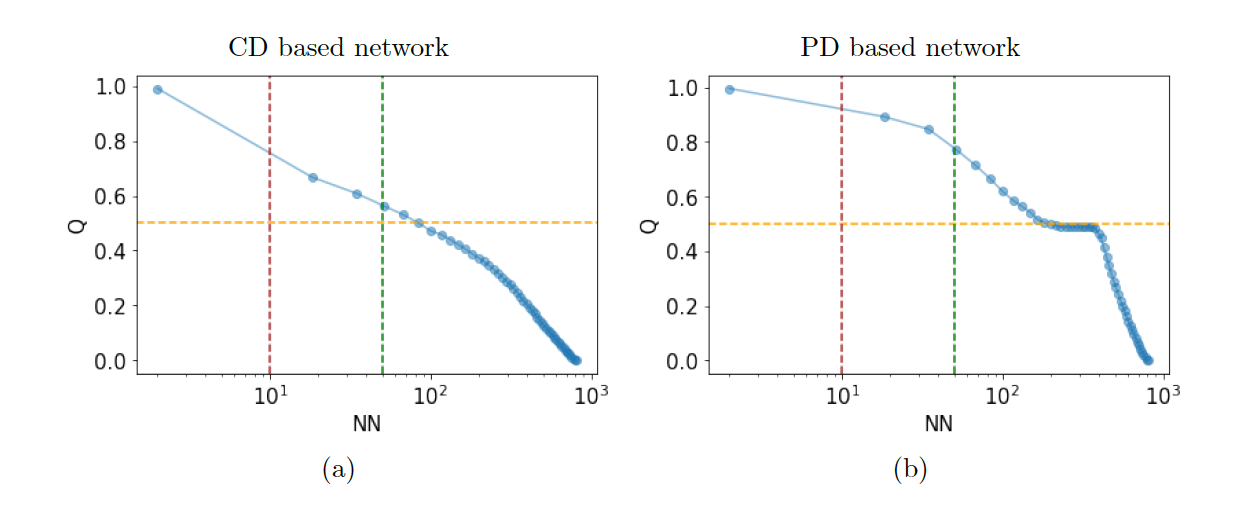

Supplement: S2 Fig — (a-b) Variation of network modularity with number of nearest neighbours (NN) in cosine distance (CD) and phylogenetic distance (PD) based networks. The vertical line mark NN = 10 and NN = 50, and horizontal line marks Q = 0.5 for (a) CD based network, and (b) PD based network. (TIF) [file pone.0287482.s002.tif]

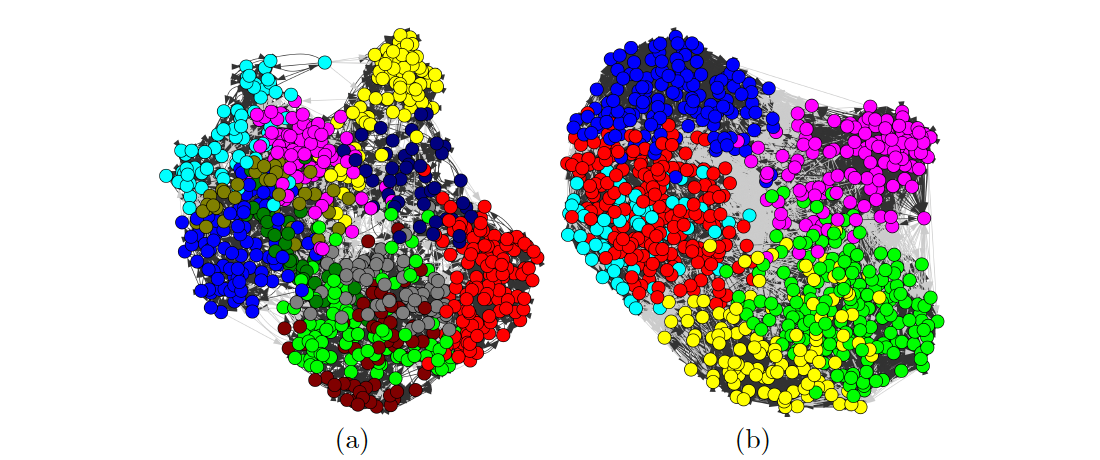

Supplement: S3 Fig — (a) The network, where each node is connected to its 10 nearest neighbors, shows 11 different modules or network communities. (b) The network where each node is connected to its 50 nearest neighbors, shows 6 modules. The colours identify different network modules. In both of these networks the directed edges within module are shown in black colour and inter-modular edges are shown with grey colour. (TIF) [file pone.0287482.s003.tif]
